# Disease Resistance Correlates with Core Microbiome Diversity in Cotton

**DOI:** 10.1007/s00284-024-03827-1

**Published:** 2024-08-08

**Authors:** Rhea Aqueel, Ayesha Badar, Nazish Roy, Umer Zeeshan Ijaz, Kauser Abdulla Malik

**Affiliations:** 1 Kauser Abdulla Malik School of Life Sciences, Forman Christian College (A Chartered University), Ferozepur Road, Lahore, 54600 Pakistan; 2 Water & Environment Research Group, Mazumdar-Shaw Advanced Research Centre, University of Glasgow, Glasgow, G11 6EW UK; 3 National University of Ireland, Galway, University Road, Galway, H91 TK33 Ireland; 4 Department of Molecular and Clinical Cancer Medicine, University of Liverpool, Liverpool, L69 7BE UK; 5 Pakistan Academy of Sciences, Islamabad, Pakistan

## Abstract

Understanding the resident microbial communities and their above and below ground interactions with plants will provide necessary information for crop disease protection and stress management. In this study, we show how diversity of core microbiome varies with disease susceptibility of a crop. To test this hypothesis, we have focused on identifying the core microbial species of cotton leaf curl disease (CLCuD) susceptible *Gossypium hirsutum* and CLCuD resistant *Gossypium arboreum* under viral infestation. Derivation of core membership is challenging as it depends on an occupancy threshold of microbial species in a sampling pool, whilst accounting for different plant compartments. We have used an abundance–occupancy distribution approach where we dynamically assess the threshold for core membership, whilst marginalizing for occupancy in four compartments of the cotton plant, namely, leaf epiphyte, leaf endophyte, rhizosphere, and root endophyte. Additionally, we also fit a neutral model to the returned core species to split them into three groups, those that are neutral, those that are selected by the plant environment, and finally those that are dispersal limited. We have found strong inverse relationship between diversity of core microbiome and disease susceptibility with the resistant variety, *G. arboreum*, possessing higher diversity of microbiota. A deeper understanding of this association will aid in the development of biocontrol agents for improving plant immunity against biotrophic pathogens.

**Supplementary Information:**

The online version contains supplementary material available at 10.1007/s00284-024-03827-1.

## Introduction

Microbes are minute but essential components of the environment which play crucial roles in the host plant’s response to disease. Insights into plant–microbe interactions have revealed a great deal about the underlying mechanisms that contribute to plant health and development [1]. Root exudates possess the ability to shape the rhizospheric microbial communities where, the composition of these exudates is genotype-dependent [2, 3]. Furthermore, there is an intricate play between plant hormones at different developmental stages, and cultivar-specific root exudate production which are influenced by the rhizospheric cotton microbiome [4].

The cotton leaf curl disease (CLCuD) is a whitefly transmitted viral disease of the cotton plant. Biotrophic pathogens, such as the one causing CLCuD, are known to increase salicylic acid (SA) levels in infected plants as this phytohormone is found to be essential for gene-for-gene resistance, systemic acquired resistance (SAR), and reduction of disease development [5]. Beneficial microbes from the phyllosphere can also switch on plant defense responses [6, 7]. Thus, plant immunity boosting non-pathogenic microbiota are the new tool for conferring disease resistance in host plants [8, 9]. Apart from the exploration of the entire microbiome, recent approaches have revealed interesting insights relating to the microbes that make up the core of plants and in turn influence the functional relationships with the host [10]. Core microbial communities are the smallest subset of stable taxa identified in plant ecosystems, that can aid the plant in nutrient and water uptake and promote plant health by activating defense responses against biotic and abiotic stresses [11].

Past attempts at identifying core microbiome membership relied on a strict occupancy/prevalence threshold [12] that varied between 50 and 95% depending on a study in consideration, which is always a source of debate. To circumvent this, Shade and Stopnisek [13] have proposed a dynamic approach where microbial species are first ranked by their occupancy according to the considered study design using both *site-specific occupancy*, and *replicate consistency* of microbial species. Starting with a seed core subset of top-ranked species, contribution of the core set to beta diversity is calculated, with species iteratively added until adding one more species offers diminishing returns on explanatory value for beta diversity. Thus, the occupancy threshold is learnt from the dataset. Furthermore, the approach is merged with Burns et al. [14] approach, where neutral model is applied to species-occupancy distribution of observed microbial species. The 95% confidence intervals of neutral models are then obtained for species plotted by mean log10 relative abundance and occupancy. The species that fall outside the 95% model are then inferred to be deterministically, rather than neutrally, selected. Overlapping the two approaches then provides a consistent methodology for prioritizing ecologically important core microbial species over space and time, and is explored in this study within the context of plant resistance against CLCuD.

## Materials and Methods

### Sample Collection

Sampling for the cotton plants was carried out at Four Brothers Research Farm (31.399043°N, 74.175621°E) and the Greenhouse at Forman Christian College University, Lahore (31.523565°N, 74.335380°E). The samples were obtained for three varieties at the vegetative stage (50 days after germination DAG) with varying susceptibility to CLCuD: *Gossypium hirsutum* susceptible variety (PFV-2); *G. hirsutum* partially tolerant variety (PFV-1); and *Gossypium arboreum* resistant variety (FDH-228). The plants exhibited disease symptoms in *G. hirsutum* 25 days post infestation. For CLCuV infection, the plants were grown under heavy viruliferous whitefly conditions. Furthermore, a total of five replicates were taken for each of the four selected plant compartments under study i.e., leaf epiphyte, leaf endophyte, rhizosphere, and root endophyte.

### DNA Isolation

Rhizospheric soil (up to 3 mm around root area) and roots of cotton plants were collected by gently shaking the roots of the plant to get rid of the bulk soil, and roots were stored in a 50 mL falcon tube. Leaves from the upper canopy showing severe CLCuD symptoms were taken for susceptible varieties. For the resistant variety, upper canopy leaves were sampled under a heavy whitefly attack to ensure cotton leaf curl virus (CLCuV) infestation. Samples were placed in an ice box until they were brought to the lab then stored at 4 °C, and the standard protocol was followed for preparation before DNA extraction within 2 days. To extract DNA from leaf epiphytes, the leaves underwent a rigorous washing process. They were washed three times with a 1× TE buffer solution that contained 0.2% Triton X. The resulting wash was collected and filtered using 0.2 µM sterile filter paper, which was then utilized for DNA extraction. For leaf endophytes, 100 mg of leaf tissue was washed rigorously with 70% ethanol, followed by 3% bleach, and several washings with sterile distilled water to eliminate leaf epiphytic bacteria. It was then ground using a pestle and mortar in PBS buffer and collected the resulting mixture in a falcon tube. Roots were sonicated in PBS buffer for 5 min to separate the closely adhered soil from the rhizospheric soil. The root was then washed rigorously with 70% ethanol, followed by 3% bleach, and several washings with sterile distilled water to eliminate rhizospheric bacteria. Eventually, the sterilized root (100 mg) was macerated in PBS buffer using a pestle and mortar and the resulting mixture was collected in a falcon tube. DNA for all four compartments (leaf epiphyte, leaf endophyte, rhizospheric soil, root endophyte) was extracted using the FastDNA™ SPIN Kit for Soil (MP Biomedicals) by following the manufacturer’s guideline. Samples were homogenized in the FastPrep instrument for 40 s at a speed setting of 6.0. The DNA was eluted in 30 µL of elution buffer.

### PCR Amplification and Sequencing

DNA sample dilutions (10 ng/µL per sample) were used for the amplification of 16S rRNA hypervariable region V3-V4 using the primers: 341F (5′-TCGTCGGCAGCGTCAGATGTGTATAAGAGACAGCCTACGGGNGGCWGCAG-3′) and 805R (5′-GTCTCGTGGGCTCGGAGATGTGTATAAGAGACAGGACTACHVGGGTATCTAATCC-3′) [15]. The choice of primer design was taken into consideration based on the benchmarking study [16]. The samples were sequenced on an Illumina MiSeq platform (Macrogen, Inc. Seoul, South Korea).

### Bioinformatics and Statistical Analysis

We have used the core microbiome approach [13] on an Amplicon Sequence Variants (ASVs) abundance table (*n* = 59 samples × *P* = 38,120 ASVs) [17] obtained after processing the samples in the QIIME 2 [18] workflow using the standard deblur software, and then assigning the taxonomy based on SILVA SSU Ref NR database v138 [19]. After pre-processing for low read samples and excluding contaminants, a total of 50 samples were retained with 34,144 ASVs with the summary statistics of ASVs per samples as follows: [1st quartile: 7979; median: 15,522; mean: 14,565; 3rd quartile: 21,387; and maximum: 27,839]. Since we are considering the four plant compartments i.e., leaf epiphyte, leaf endophyte, rhizosphere, and the root endophyte, therefore we have used the compartment-specific occupancy model.

**Fig. 1:**
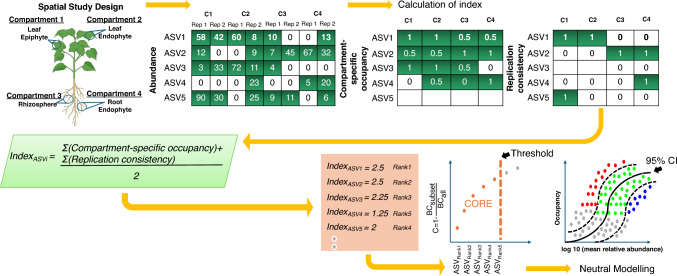
Graphical illustration of core microbiome strategy

On the abundance table, along with the categorical information of these compartment, we have applied the core microbiome analysis [13]. This is a better suited model for inferring core microbiome as it frees one from choosing a crisp threshold (typically 50% or 85% prevalence for defining the core microbiome). The approach considers sample occupancy of ASVs across sites along with the replicate information, and then calculates the minimal occupancy threshold dynamically by learning from the data. The ranking of ASVs is done using a combination of two metrics: site-specific occupancy (ASVs occupation in the four compartments namely, Leaf Epiphyte, Leaf Endophyte, Rhizosphere, and Root Endophyte); and replicate consistency (consistency of ASVs across replicates within each compartment). The core microbiome analysis was done separately for each of the three varieties, FDH-228, PFV-1, and PFV-2, respectively. After ranking the ASVs, the subset of core taxa is constructed by iteratively adding one ASV at a time to the core set of ASVs, from highly ranked ASVs to the lowly ranked ones. The contribution of the core subset to beta diversity is then calculated every time a new ASV becomes member of the core set using the Bray–Curtis contribution, $C=1-\frac{{\text{BC}}_{\text{core}}}{{\text{BC}}_{\text{all}}}.$ There are two stopping criteria used in [13], of which we have used the following relaxed criteria (as per recommendation by original authors) at which incorporation of the new ASV in the core subset should stop: *addition of an additional ASV does not cause more than 2% increase in the explanatory value by Bray–Curtis distance*. Independently, the neutral model [14] is fitted to the abundance–occupancy distributions of the ASVs. As a result, the subset of these ASVs which belong to core subset are further categorized into three subsets: (a) those that satisfy the 95% confidence interval of the neutral model, and are driven by stochastic processes; (b) those that fall above the 95% confidence of the neutral model and are selected by the host environment (variety in this case); and (c) those that fall below the model, and are driven by dispersal limitation process. The whole process is shown in Fig. 1. The taxonomy tree of the core microbiome across different varieties and compartments were then drawn using the R’s metacoder package [20].

## Results and Discussion

**Fig. 2:**
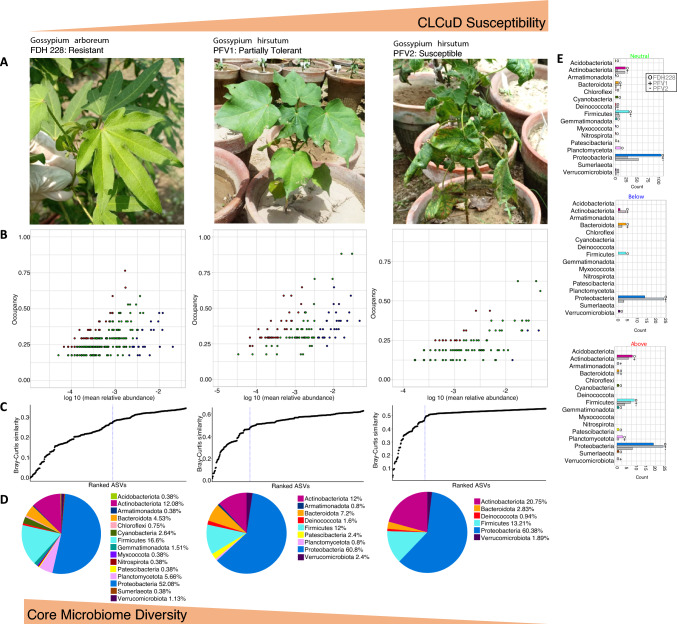
Core microbiome for three CLCuD varieties are shown in (**a**) and identified through species-occupancy abundance diagrams in (**b**) incorporating a *Plant Compartment Specific Occupancy* model (compartments being *Leaf Epiphyte*, *Leaf Endophyte, Rhizosphere*, and *Root Endophyte*). The blue dotted line in (**c**) represents the “Last 2% decrease” criteria where ASVs are incorporated in the core subset until there is no more than 2% decrease in beta diversity. (**d**) The Phylum level assignment of the ASVs. Independently a neutral model is fitted with those ASVs that fall within the 95% confidence interval, shown in green in (**b**), and those that fall outside the 95% model confidence to be inferred as deterministically assembled, i.e., non-neutral ASVs. Points above the model are selected by the host environment, shown in red in (**b**), and points below the model are dispersal limited, shown in blue in (**b**). The count of neutral/non-neutral ASVs at Phylum level are shown with the bar plots in (**e**)

**Fig. 3:**
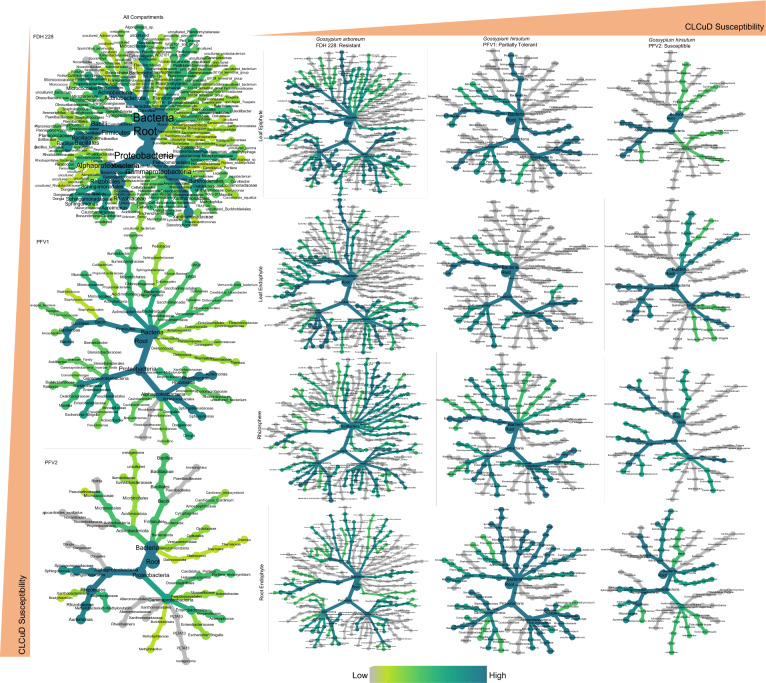
Taxonomic coverage of core microbiome in CLCuD susceptible and tolerant varieties, as calculated in Fig. 1. Left (all compartments) right (separate coverage in different compartments: leaf epiphyte, leaf endophyte, rhizosphere, and root endophyte)

At the heart of complex microbial assemblages lies its stable and shared subset of taxa termed as the ‘core microbiome’. Through omics approaches, the concept of the core microbiome can be extended beyond taxonomically defined membership to include community function and behaviour which can provide deeper ecological insights into core microbiomes [21]. The core microbiome has differentiating patterns in terms of disease resistance exhibiting higher diversity in the CLCuD resistant FDH-228 as compared to the partially tolerant PFV-1 and susceptible PFV-2. The distinctive phyla such as Sumerlaeota, Myxococcota, Gemmatimonadota, Patescibacteria, Deinococcota, Chloroflexi and Nitrospirota were identified in the core microbiome of FDH-228. The resultant core microbiome is shown in Figs. 2 and 3. Our findings support that CLCuD resistant variety associated microbiome is dynamic and is therefore selected by the cotton species itself due to its underlying functional relationship with the host plant. Despite their significance, the removal of a core member does not always lead to the collapse of the ecosystem due to the redundant functions that other microbes can perform. Therefore, the presence of a core member is vital, but not always indispensable for the survival of the community [22].

Our targeted sequencing effort gives a precise insight into the core microbial communities of the susceptible, partially tolerant, and resistant cotton varieties infected with CLCuV. On the phylum level, the different plant species under CLCuD attack had specific effects on the bacterial communities. The core taxa exhibited varying shifts between *G. hirsutum* and *G. arboreum*. Although, there were a few ASVs shared between the microbiomes, however, phyla such as Sumerlaeota and Gemmatimonadota which are known to thrive in harsh environments, were only identified in the CLCuD resistant FDH-228. Members of Gemmatimonadota are known to withstand harsh environments such as saline soils and form a very small fraction of the bacterial community [23]. The plant susceptibility to viruses is exhibited in the diversity of the core microbiome. Patescibacteria were linked with the partially tolerant PFV-1 and resistant FDH-228. A study found Acidobacteriota in the core taxa of the interspecific interaction zone of peanut and sorghum rhizosphere [24]. Acidobacteriota has only been identified in the core microbial taxa of the CLCuD resistant FDH-228. After fitting the neutral model, as compared to the susceptible PFV-2 and partially tolerant PFV-1, we have found the resistant variety FDH-228 to be selecting more for Actinobacteriota, Cyanobacteria, Firmicutes, Gemmatimonadota, Patescibacteria, Planctomycetota, and Sumerlaeota. Actinobacteriota, Patescibacteria, and Planctomycetota, have been reported in the root endosphere of *Myrothamnus flabellifolia* which can withstand extreme drought conditions [25].

The selection of core microbes by the host plant is a fundamental process that ensures efficient colonization and is critical in enhancing the ability of these microbes to thrive and interact with the plant’s environment. Therefore, it is necessary to demonstrate the importance of microbes that have been deterministically selected by the host. In this paper, we have identified the core microbes at the genus level (Supplementary_Data_Table_2), particularly those that are fitted above the neutral model and are selected by the host plant itself. The shared microbial taxa between FDH-228 and PFV-1 include *Bacillus*, *Cutibacterium*, *Sphingomonas*, *Dongia*, *Methylobacterium-Methylorubrum*, *Staphylococcus*, *Massilia*, *Micrococcus* and *Escherichia-shigella*. The ones that are selective to the resistant variety FDH-228 include *Aureimonas*, *Aquabacterium*, *Saccharimonadales*, *Stenotrophomonas*, *Streptomyces*, *Kocuria*, *Arenimonas*, *Methylophilus*, *Nocardiodes*, *Rhodospirallales*, and *Paenisporosarcina*. The presence of rare microbiota in the host’s system exemplifies a significant bacterial reservoir, offering a plethora of physiological and ecological attributes. Certain rare microbial species such as *Cutibacterium*, *Kocuria* and members of the family *Comamanadaceae* were found as core endophytic bacteria in Jasione plants and contribute significantly to its diversity and tolerance against arsenic stress [26]. The bacterial genera selected by the PFV-1 plant include *Acidibacter*, *Fimbriimonadaceae*, *Ferrovibrio*, *Verrucomicrobia bacterium*, *Devosiaceae*, *Comamonadaceae*, *Paracoccus*, *Brevundimonas*, *Enterbactericeae*, and *Gemmataceae.* PFV-2 harbors *Arsenophonus* and *Cardinium symbiont* whereas *Bacillus*, *Acinetobacter*, *Sphingomonas*, and *Methylobacterium-Methylorubrum* are found in both the selected *G. hirsutum* varieties: PFV-2; and PFV-1. *Rhizobiaceae* and *Aureimonas* are shared between FDH-228 and PFV-2. *Acinetobacter* and *Aureimonas* as components of the rice phyllospheric core microbiome have been reported to confer resistance against blast disease upon foliar spray application [27].

CLCuD is one of the major biotic stresses in Pakistan causing severe economic losses up to more than US$2 billion per annum [28] in cotton crops, and with Pakistan being one of the top cotton producers, any strategy to suppress CLCuD will have significant impacts. The observed symptoms of CLCuD include vein thickening, leaf curling, leaf enation and dwarfing in the highly susceptible cotton genotypes eventually lead to mortality of the plant [29–32]. There is very little [17] or no evidence to suggest that the use of plant-associated microbiome will lead to suppression of CLCuD. The diploid species *G. arboreum* has resistance genes against CLCuD, but the widely cultivated *G. hirsutum* is susceptible to the disease. Treatment with the members of core microbial community inferred from the *G. arboreum* may hold the key to safeguard *G. hirsutum* which is cultivated > 90% worldwide [33]. Rhizospheric core microbial communities are previously known to offer promising plant disease resistance [34, 35], albeit for *Arabidopsis*. Nonetheless, the construction of synthetic communities (SynComs) remains challenging and depends on several factors including the spatial and temporal dynamics and on the coordination of the microbes forming the community [36, 37].

## Conclusion

Through this research, and using a dynamic inferential approach, we anticipate that the obtained core microbiome of *G. arboreum* (FDH-228: resistant) and its diversity will aid in developing SynComs and may offer biocontrol potential by altering the plant–microbe ecology in the CLCuD infected cotton plant, thereby presenting future microbiome-mediated driven solutions for sustainable agriculture.

### Supplementary Information

Below is the link to the electronic supplementary material.

Supplementary file1 The meta data associated with the deposited sequences (XLSX 13 KB)

Supplementary file2 The detailed dataset for Figures 2 and 3 (XLSX 41 KB)

## Data Availability

The raw sequence files supporting the results of this article are available in the European Nucleotide Archive under the project accession number PRJEB67645 with details of the samples provided in Supplementary_Data_Table_1.xlsx.

## Code Availability

Some of the code used to generate the results are part of microbiomeSeq (https://github.com/umerijaz/microbiomeSeq), whilst other codes are available at http://userweb.eng.gla.ac.uk/umer.ijaz#bioinformatics.

## Declarations

### Deposition in Repositories

Yes, the sequencing data are deposited in public repository.
